# Serotype 1 and 8 Pneumococci Evade Sensing by Inflammasomes in Human Lung Tissue

**DOI:** 10.1371/journal.pone.0137108

**Published:** 2015-08-28

**Authors:** Diana Fatykhova, Anne Rabes, Christoph Machnik, Kunchur Guruprasad, Florence Pache, Johanna Berg, Mario Toennies, Torsten T. Bauer, Paul Schneider, Maria Schimek, Stephan Eggeling, Timothy J. Mitchell, Andrea M. Mitchell, Rolf Hilker, Torsten Hain, Norbert Suttorp, Stefan Hippenstiel, Andreas C. Hocke, Bastian Opitz

**Affiliations:** 1 Department of Internal Medicine/Infectious Diseases and Pulmonary Medicine, Charité—Universitätsmedizin Berlin, Augustenburger Platz 1, 13353, Berlin, Germany; 2 Bioinformatics, Centre for Cellular and Molecular Biology, Hyderabad, Telangana, India; 3 Lungenklinik Heckeshorn, HELIOS Klinikum Emil von Behring, Walterhöferstrasse 11, 14165, Berlin, Germany; 4 Department for General and Thoracic Surgery, DRK Clinics, Drontheimer Strasse 39–40, 13359, Berlin, Germany; 5 Vivantes Klinikum Neukölln, Department for Thoracic Surgery, Berlin, Rudower Straße 48, 12351, Berlin, Germany; 6 Institute of Microbiology and Infection, School of Infection and Immunity, University of Birmingham, Birmingham, B15-2TT, United Kingdom; 7 Institute of Medical Microbiology, Justus-Liebig University Giessen, Schubertstrasse 81, D-35392, Giessen, Germany; 8 Department of Bioinformatics and Systems Biology, Justus-Liebig University Giessen, Heinrich-Buff-Ring 58, D-35392, Giessen, Germany; Centers for Disease Control & Prevention, UNITED STATES

## Abstract

*Streptococcus pneumoniae* is a major cause of pneumonia, sepsis and meningitis. The pore-forming toxin pneumolysin is a key virulence factor of *S*. *pneumoniae*, which can be sensed by the NLRP3 inflammasome. Among the over 90 serotypes, serotype 1 pneumococci (particularly MLST306) have emerged across the globe as a major cause of invasive disease. The cause for its particularity is, however, incompletely understood. We therefore examined pneumococcal infection in human cells and a human lung organ culture system mimicking infection of the lower respiratory tract. We demonstrate that different pneumococcal serotypes differentially activate inflammasome-dependent IL-1β production in human lung tissue and cells. Whereas serotype 2, 3, 6B, 9N pneumococci expressing fully haemolytic pneumolysins activate NLRP3 inflammasome-dependent responses, serotype 1 and 8 strains expressing non-haemolytic toxins are poor activators of IL-1β production. Accordingly, purified haemolytic pneumolysin but not serotype 1-associated non-haemolytic toxin activates strong IL-1β production in human lungs. Our data suggest that the evasion of inflammasome-dependent innate immune responses by serotype 1 pneumococci might contribute to their ability to cause invasive diseases in humans.

## Introduction

*Streptococcus pneumoniae* is both a frequent colonizer of the human nasopharynx and a major cause of invasive diseases. Depending on preceding viral infections, the immune status of the host, and on the pneumococcal strain, an asymptomatic colonization can establish and progress to pneumonia, sepsis, or meningitis [1,2]. These infections are associated with high morbidity and mortality. It is estimated that *S*. *pneumoniae* causes over 1 million infant deaths every year worldwide and probably even more in elderly people and immunocompromised patients [3].

The more than 90 capsular serotypes of *S*. *pneumoniae* vary markedly in their ability to cause invasive infection. For example, serotypes 3, 6B, 9N and others are associated with nasopharyngeal colonization but also with infections in patients with underlying diseases, and with higher mortality when causing pneumonia. In contrast, dominating clones of serotypes 1 and 8 are frequently found in invasive diseases that, however, show lower case-fatality rates [4–7]. Among serotype 1 pneumococci, the recently emerging MLST306 clone now dominates by over 80% in many parts of the world [8–13]. While the degree of encapsulation positively associates with colonization prevalence and virulence [5], other bacterial factors that affect the interaction with the host´s innate immune system might also play a role.

In addition to the capsule, pneumolysin (PLY) is a major virulence factor of *S*. *pneumoniae* [1,14]. PLY is a member of the cholesterol-dependent cytolysins expressed by various Gram-positive bacteria. PLY of most pneumococci binds to cholesterol-containing membranes, forms pores upon oligomerization, and thereby causes cell lysis [15,16]. At sublytic concentrations, PLY has been described to activate the complement system and to stimulate cytokine production in monocytes and macrophages [1,14]. Moreover, PLY has been implicated in biofilm formation, independent of its haemolytic activity [17]. Studies in mouse models of primary pneumococcal pneumonia demonstrated that PLY-deficient strains are more rapidly cleared from the lungs and induce less inflammation compared to isogenic toxin-producing bacteria [18–20]. Interestingly, PLY exists in at least 16 different protein variants with variable haemolytic activity. For example, allele 5 PLY expressed by serotype 1 MLST306 and some serotype 8 pneumococci is non-haemolytic in contrast to the allele 1 PLY of the D39 strain [9,21,22].

Recent studies by us and others showed that the innate immune system can sense haemolytic PLY through the canonical NLRP3 inflammasome [23–26]. Canonical inflammasomes are multiprotein complexes mainly expressed in monocytes and macrophages. They are composed of a receptor molecule belonging to the NOD-like receptor family (such as NLRP3) or the PYHIN protein family (e.g. AIM2), the adapter molecule ASC and caspase-1 [27,28]. Inflammasomes post-transcriptionally regulate the production of IL-1β and IL-18 by a caspase-1-dependent processing of the cytokine pro-forms into mature cytokines, and induce an inflammatory cell death called pyroptosis. Mice lacking inflammasome components or receptors for IL-1 or IL-18 show an altered susceptibility towards *S*. *pneumoniae* [23–26,29,30], indicating that inflammasomes are critical components of the innate defence system during pneumococcal infection.

Here we demonstrate that different pneumococcal strains are differentially recognized by the innate immune system in human lung tissue. The NLRP3 inflammasome senses *S*. *pneumoniae* expressing haemolytic PLY in human lung tissue, whereas the pneumococcal serotypes 1 and 8 expressing non-haemolytic toxins are poor inflammasome activators.

## Materials and Methods

### Bacterial strains and PLYs

*S*. *pneumoniae* serotype 2 D39 and D39Δ*ply* have been described before [31,32]. *S. pneumonia*e serotype 1 multi locus sequence type (MLST)306, serotype 3 MLST180, serotype 6B MLST176, serotype 8 MLST53 and serotype 9N MLST66 were kindly provided by the National Reference Centre for Streptococci, Germany. Bacteria were grown in THY media at 37°C and 5% CO_2_ until they reached a phase of logarithmic growth. The PLY gene was amplified by PCR from the appropriate strain and cloned into pET33b (Novagen) which results in addition of poly-His tag to the N-terminus of the protein. The resulting construct was transformed into *E*. *coli* BL21 for expression following manufactures instructions. Cell extracts were prepared by sonication of cell pellets followed by clarification by centrifugation. PLY was purified by metal affinity purification of the His-tagged protein following the manufacturer’s instructions. Purity was confirmed by Coomassie Blue staining of proteins separated by SDS-PAGE.

### Cells and infection

Human PBMCs were purified from buffy coats by gradient centrifugation as described previously [33]. Cells were infected with 1 x 10^6^ (MOI = 1) or 1 x 10^4^ (MOI = 0.01) CFU/mL *S*. *pneumoniae* or treated with 10 μM Z-YVAD (Merck Millipore), allele 1 or allele 5 PLY (0.25, 0.5, 1 μg/ml).

### Human lung tissue

Lung tissue samples were obtained from 33 patients primarily suffering from bronchial carcinoma, which underwent lung resection at local thoracic surgeries, and prepared as described previously [34,35]. Written informed consent was approved by all patients and the study was approved by the ethic committee at the Charité clinic (protocol number EA2/050/08 and EA2/023/07). For infection tumor-free normal lung tissue was injected with 200 μl of *S*. *pneumoniae* 10^6^ CFU/mL per 100 μg tissue for 24 hours or treated with 100μM glybenclamide (Sigma-Aldrich, St. Louis, MO, USA) 2 h before infection.

### ELISA

IL-1β and IL-8 release was quantified by ELISA in cell-free and tissue-free supernatants (eBioscience; BD Biosciences). Lactate dehydrogenase (LDH) release was quantified by CytoTox 96 Non-Radioactive Cytotoxicity Assay (Promega).

### Quantitative PCR

For analysis of transcriptional *Il1b* regulation, total cellular RNA was isolated, transcribed to cDNA, and amplified by quantitative RT-PCR using Gene Expression Master Mix and *Il1b* TaqMan Gene Expression Assay (Applied Biosystems).

### Haemolytic assay

Bacteria were lysed for 1 h at 4°C (5 mg/mL lysozyme (Sigma-Aldrich), 25 mM Tris-HCl, 50 mM NaCl, protease inhibitor cocktail (Roche)) followed by sonication. The cellular fraction of human blood was PBS-washed, diluted in PBS (2%) and incubated with pneumococcal lysates for 1 h at room temperature. After centrifugation, red blood cell lysis was assessed. The highest amount of pneumococcal lysate used were generated from 5x10^6^ CFU *S*. *pneumonia*e. Adjacent wells in a row represent a 2-fold dilution.

### Western blot

Proteins from human lung tissue were extracted using lysis buffer and disrupted in FastPrep-24 homogenizer. Anti-IL-1beta, actin (Santa Cruz Biotechnology) and anti-NLRP3 (Adipogen) antibodies were used. Proteins were detected by incubation with HRP-conjugated IgG antibodies (Santa Cruz Biotechnology) and Pierce ECL Western Blotting Substrate (Thermo Scientific).

### Comparative protein modelling of PLY

Three-dimensional protein structure of allele 5 PLY was built by comparative modelling using the MODELLER 9.11 software [36]. The crystal structure of perfringolysin O (PDB: 1PFO) was used as a template for modelling [37].

### Sequencing of bacterial strains

*S*. *pneumoniae* serotypes (1, 3, 6B, 8 and 9N) were genome sequenced as described below using Illumina’ MiSeq next generation sequencing system. Chromosomal DNA was isolated using PureLink Genomic DNA Mini Kit (Life Technologies). Nextera XT paired-end library was prepared and sequenced on a MiSeq using v3 chemistry, according to protocols recommended by the manufacturer (Illumina). Sequencing reads were assembled using SPades [38] and contigs were ordered by r2cat [39] using *S*. *pneumoniae* D39 genome (accession number NC_008533) as reference genome. Draft genome sequences were submitted to the GenDB annotation system [40]. Finally, pneumolysin DNA sequences for each *S*. *pneumoniae* serotype were provided by GenDB, whereas draft genome sequence reads will be used for further comparative genomic analysis.

### Data Analysis

Data are expressed as mean ± SEM. Samples were tested for a normal distribution with the D’Agostino and Pearson Omnibus Normality Test. Statistical analysis of normally distributed samples was performed using ANOVA followed by a Bonferroni post-hoc test and samples that were not normally distributed were analysed with Kruskal–Wallis test followed by Dunn’s multiple comparison test. The Mann-Whitney U Test was used for the comparison of two populations. Data analysis was performed using the Prism software (GraphPad Software, La Jolla, CA). For all statistical analyses, p values < 0.05 were considered significant: *p < 0.05, **p < 0.01, ***p < 0.001.

## Results

*S*. *pneumoniae* serotypes with variable haemolytic activities differentially stimulate caspase-1-dependent production of IL-1β by human cells.

**Fig 1:**
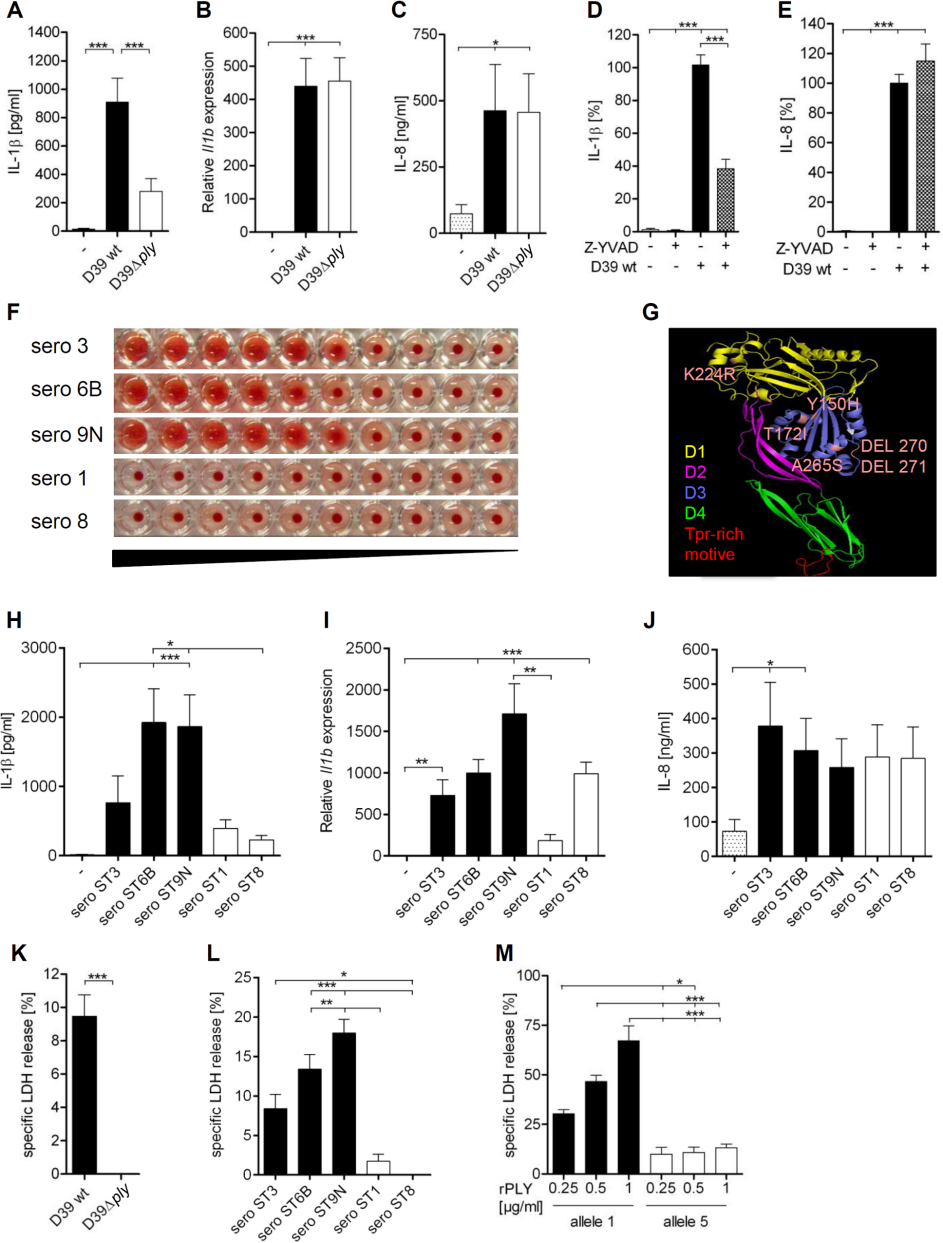
*S*. *pneumoniae* serotypes differentially induce PLY- and caspase-1-dependent production of IL-1β by PBMCs as well as cell death. (A-C, H-L) PBMCs were infected with *S*. *pneumoniae* strains (MOI = 1) as indicated or (D, E) were infected with *S*. *pneumoniae* D39 (MOI = 0.01) and treated with 10 μM Z-YVAD. Production of IL-1β (A, D, H) and IL-8 (C, E, J) was quantified by ELISA after 16 h. (D, E) 100% were defined as the amount of IL-1β or IL-8 released by *S*. *pneumoniae*-infected PBMCs. (B, I) Relative expression of *Il1b* was determined by quantitative RT-PCR after 5 h. (F) Human blood was incubated with pneumococcal lysates of *S*. *pneumoniae* serotypes 3, 6B, 7F, 9N, 1 and 8, and haemolytic activity was assessed. (G) Comparative protein model of allele 5 PLY. PLY domains are colour coded and mutations are marked in rose. (K) PBMCs were infected with *S*. *pneumoniae* D39 and D39Δ*ply*. (L) PBMCs were infected with serotypes 3, 6B, 7F, 9N, 1 and 8 pneumococci. (M) PBMCs were stimulated with allele 1 and allele 5 PLY for 16 h. (K-M) LDH release was quantified by cytotoxicity assay. Data are shown as mean ± SEM of three (D, E, M), five (B, I) or seven (A, C, H, J, K, L) independent experiments carried out in duplicates or triplicates. Significance is indicated by asterisks * = p<0.05, ** = p<0.01, *** = p<0.001.

To confirm that production of IL-1ß by human cells is dependent on PLY and on conventional inflammasomes, we infected human PBMCs with *S*. *pneumoniae* serotype 2 expressing haemolytic allele 1 PLY (D39) or with the isogenic mutant lacking PLY (D39Δ*ply*) and tested the effect of a caspase-1 inhibitor. We found that only *S*. *pneumoniae* expressing PLY stimulated strong IL-1β production, whereas the PLY-negative mutant induced only small amounts of IL-1β (Fig 1A). In contrast, transcriptional regulation of *Il1b* mRNA and production of the inflammasome-independent chemokine IL-8 was not affected by the lack of PLY (Fig 1B and 1C). Moreover, IL-1β but not IL-8 secretion was strongly reduced by treatment with the caspase-1 inhibitor Z-YVAD (Fig 1D and 1E). These data are in line with previously published studies and show that pneumococcal PLY is recognized by a conventional caspase-1-dependent inflammasome to stimulate IL-1β production [23,24].

Next, we investigated the ability of different pneumococcal strains to induce haemolysis and IL-1β production. *S*. *pneumoniae* serotypes 3, 6B, and 9N induced red blood cell lysis, indicating expression of a haemolytic PLY (Fig 1F). In contrast, serotypes 1 and 8, expressing a non-haemolytic PLY, exhibited no haemolytic activities, as shown before (Fig 1F) [21]. Sequencing of the *ply* gene confirmed the expression of an allele 5 PLY in serotype 1 and 8 bacteria, and demonstrated that serotype 3, 6B and 9N express haemolytic allele 1 or 2 PLY (Table 1). Allele 5 PLY possesses several mutations in the protein domain D3 (Fig 1G), which is suggested to insert into the host cell membrane after conformational changes and is therefore crucial for the pore-forming ability of PLY [41]. In accordance, only serotype 6B and 9N stimulated a strong IL-1β production in human PBMCs, whereas serotypes 1 and 8 did not (Fig 1H). Unexpectedly, serotype 3 was also a rather weak inducer of IL-1β production. This suggests that other factors than PLY, such as perhaps an extensive capsule production of serotype 3 pneumococci, affect the innate sensing of the bacteria. All tested bacteria induced the transcriptional regulation of *Il1b* mRNA (Fig 1I) and equally stimulated production of the inflammasome-independent chemokine IL-8 (Fig 1J). Moreover, cell death induced by *S*. *pneumoniae* D39 was dependent on PLY (Fig 1K), and correlated with expression of haemolytic PLY (Fig 1L). Accordingly, purified allele 1 PLY induced a much stronger cell death as compared to allele 5 PLY in humans cells (Fig 1M). Collectively, the data confirm and extend previously published results [24], and demonstrate that pneumococcal strains expressing diverse toxins induce different amounts of IL-1β production and cell death.

**Table 1:** Pneumolysin allele determination by sequencing of *ply* genes was performed from the 6 isolates used in this study. The first row refers to amino acid positions. The second row shows allele 1 PLY expressed in *S*. *pneumoniae* D39 [21]. The amino acid polymorphisms in the clinical isolates are highlighted. Deletion of an amino acid is represented by the abbreviation DEL. Serotype 1 and 8 pneumococci express an allele 5 PLY as previously shown [21].

Serotype	MLST	Allele	Amino acid position						
			150	172	224	265	270	271	380
2 (D39)	MLST128	**1**	Y	T	K	A	V	K	D
6B	MLST176	**1**	Y	T	K	A	V	K	D
1	MLST306	**5**	**H**	**I**	**R**	**S**	**DEL**	**DEL**	D
8	MLST53	**5**	**H**	**I**	**R**	**S**	**DEL**	**DEL**	D
3	MLST180	**2**	Y	T	K	A	V	K	**N**
9N	MLST66	**2**	Y	T	K	A	V	K	**N**

### The production of IL-1β in *S*. *pneumoniae*-infected human lung tissue is dependent on PLY and caspase-1

**Fig 2:**
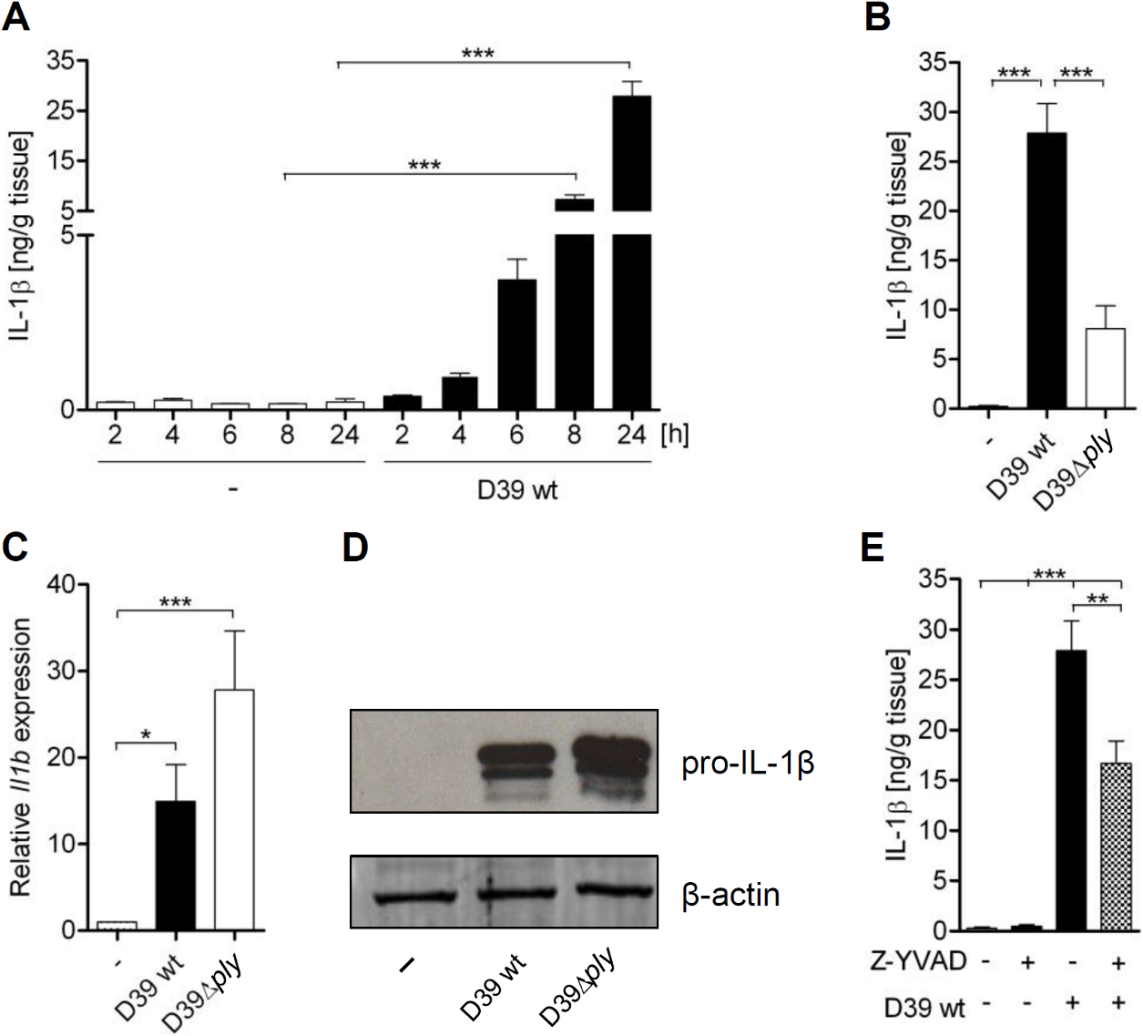
The production of IL-1β in *S*. *pneumoniae*-infected human lung tissue is dependent on PLY and caspase-1. (A-E) Human lung tissue was infected with 1x10^6^ CFU/mL *S*. *pneumoniae* serotype 2 D39 or D39Δ*ply* and treated with 100 ng/ml Z-YVAD 1 hour before infection where indicated. After 24 h, production of IL-1β (A, B, E) was quantified by ELISA, relative expression of *Il1b* was determined by qRT-PCR (C), or expression of pro-IL-1β was assessed by immunoblotting (D). Data are shown as mean ± SEM of three (A), five (E) or six (B, C) independent experiments carried out in duplicates. (D) One representative of four independent experiments is shown. Significance is indicated by asterisks ** = p<0.01, *** = p<0.001.

Next, we tested if the *S*. *pneumoniae*-induced production of IL-1β in human lungs is also dependent on PLY and caspase-1. We found that *S*. *pneumoniae* D39 but not D39Δ*ply* stimulated strong IL-1β production in human lung tissue (Fig 2A and 2B). In contrast, both strains induced expression of *Il1b* mRNA and proIL-1β protein (Fig 2C and 2D). Inhibition of caspase-1 by the specific inhibitor Z-YVAD reduced the pneumococci-induced production of IL-1β (Fig 2E). Thus, production of IL-1β in *S*. *pneumoniae*-infected human lung tissue is dependent on PLY and inflammasomes.

### The polymorphism in PLY affects production of IL-1β in human lung tissue

**Fig 3:**
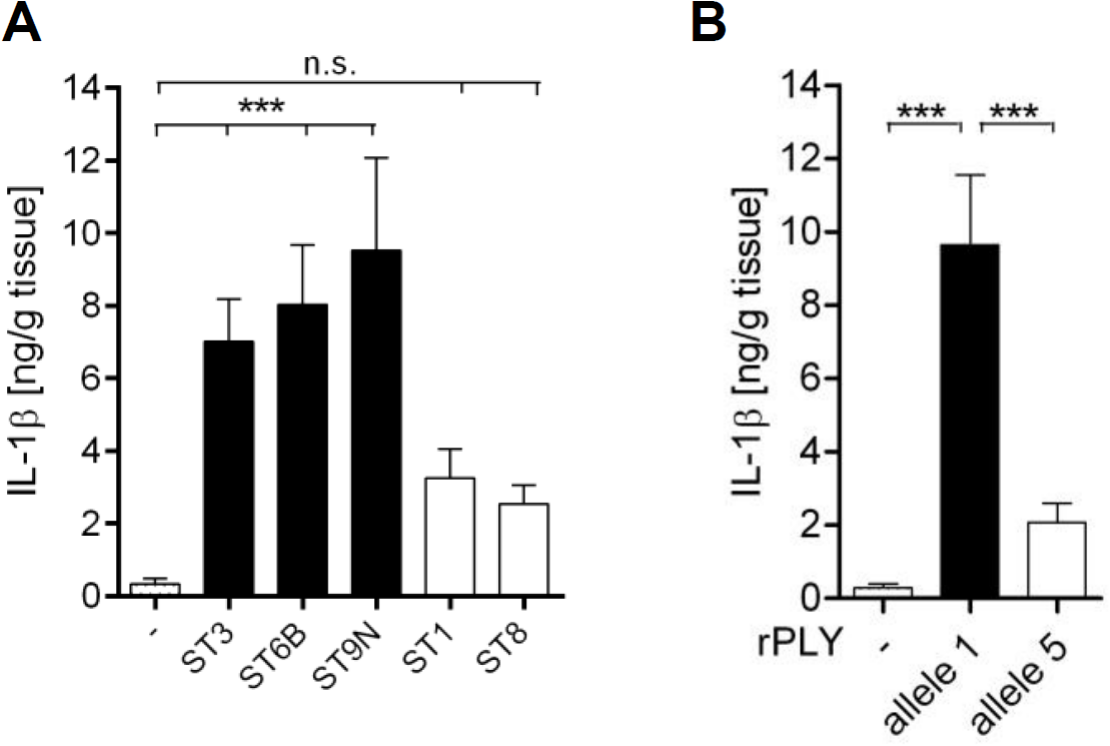
Pneumococci expressing haemolytic but not non-haemolytic PLY stimulate IL-1β production in human lung tissue. (A) Human lung tissue was infected with 1x10^6^ CFU/mL *S*. *pneumoniae* serotypes 3, 6B, 9N, 1 and 8, or (B) were stimulated with 1μg/ml allele 1 and allele 5 PLY for 24 h. IL-1β secretion was quantified by ELISA. Data are shown as mean ± SEM of eight (A) or six (B) independent experiments carried out in duplicates. Significance is indicated by asterisks *** = p<0.001.

We then infected human lung tissue with different haemolytic (serotypes 3, 6B, 9N) and non-haemolytic (serotypes 1, 8) pneumococcal strains. We found that only the haemolytic *S*. *pneumoniae* strains significantly induced IL-1β production in human lungs, whereas the non-haemolytic serotype 1 and 8 bacteria did not (Fig 3A). Accordingly, haemolytic allele 1 PLY stimulated a much stronger IL-1β production in human lungs as compared to non-haemolytic allele 5 toxin (Fig 3B). These data indicate that the polymorphism in PLY also affects production of IL-1β in human lung tissue. The serotype 1 and 8 bacteria expressing a non-haemolytic PLY appear to evade recognition by inflammasomes, and therefore induce little IL-1β production.

### Production of IL-1β in *S*. *pneumoniae*-infected human lungs is dependent on NLRP3

**Fig 4:**
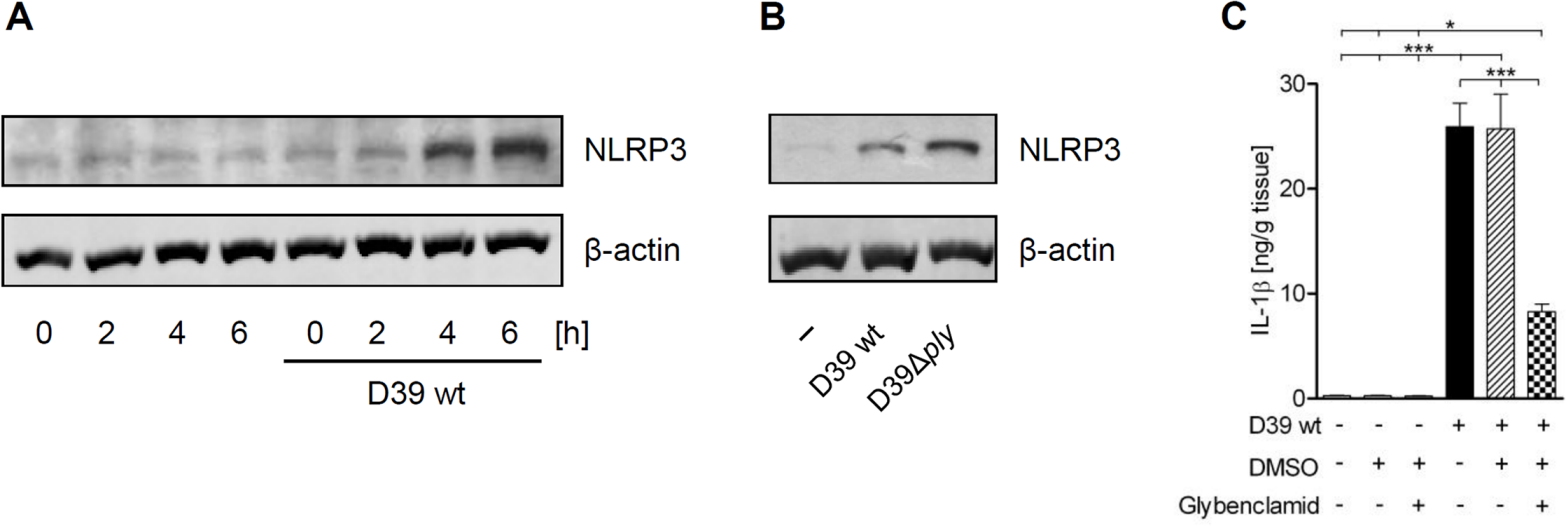
NLRP3 expression is up-regulated in human lung tissue during *S*. *pneumoniae* infection. (A, B) Human lung tissue was infected with 1x10^6^ CFU/mL *S*. *pneumoniae* serotype 2 D39 or D39Δ*ply* and expression of NLRP3 was assessed by immunoblotting. (C) NLRP3 inhibitor glybenclamide efficiently reduced IL-1β secretion in human lung tissue during *S*. *pneumoniae* infection. Human lung tissue was preincubated with the inhibitor for 2 h and infected with 1x10^6^ CFU/mL *S*. *pneumoniae* D39 for 16 h. IL-1β production was quantified by ELISA. Data are shown as mean ± SEM of three (A, B) or six (C) independent experiments carried out in duplicates. Significance is indicated by asterisks *** = p<0.001.

Finally, we tested if IL-1ß production in *S*. *pneumoniae*-infected human lung tissue is dependent on NLRP3. We first checked expression of NLRP3 in the tissue and found up-regulation of NLRP3 upon *S*. *pneumoniae* D39 and D39Δ*ply* infection (Fig 4A and 4B). Second, we treated the lung tissue with the inhibitor Glibenclamid. Glibenclamid blocks potassium channels that are required for NLRP3 inflammasome activation [42], and is therefore frequently used as a specific NLRP3 inflammasome inhibitor [43]. We found that inhibition of NLRP3 strongly reduced the *S*. *pneumoniae*-induced IL-1β production (Fig 4C). Together, these data indicate that the NLRP3 inflammasome mediates IL-1β production upon *S*. *pneumoniae* infection by detecting haemolytic PLY in human lung tissue.

## Discussion

The NLRP3 inflammasome is an important mediator of innate immunity during pneumococcal pneumonia. It senses pneumococcal PLY in murine macrophages and mediates production of the key inflammatory cytokines IL-1β and IL-18 [23,24]. Accordingly, mice deficient in inflammasome components or receptors for IL-1 or IL-18 show an altered susceptibility towards *S*. *pneumoniae* [23–25,29,30]. Although rodents, and in particular mice, are widely used in biomedical research, increasing evidence suggests that some mechanisms and pathways of the immune system differ between rodents and humans. It is therefore important to prove the relevance of mechanisms that have been identified in murine models in human-relevant systems. In this study, we used an *ex vivo* explant model allowing for the investigation of bacterial [34] and viral infections in human lung tissue [44,45]. We demonstrate that the detection of PLY by the NLRP3 inflammasome is also critically involved in the production of IL-1ß in human lung tissue. Furthermore, various pneumococcal strains expressing natural PLY variants differentially induce inflammasome-dependent IL-1β production by human cells and lung tissue.

PLY is a major virulence factor of *S*. *pneumoniae* that exhibits a wide variety of activities consistent with virulence [14–16,46,47]. It is cytotoxic to host cells, inhibits ciliary beating on the respiratory epithelium, activates the classical complement system, and stimulates inflammasomes [14]. Accordingly, bacterial mutants lacking PLY have been shown to be more rapidly cleared from the lungs compared to wild-type bacteria [18–20]. It is, however, less clear which of the different activities of PLY contribute most to pneumococcal virulence.

The MLST306 serotype 1 clone has emerged as being responsible for over 80% of serotype 1 diseases in many parts of the world, and has been associated with invasive pneumococcal disease outbreaks [9,10,12,13]. MLST306 pneumococci express allele 5 PLY which differs in a few amino acids from haemolytic allele 1 PLY and lacks any haemolytic activity but retains the ability to bind to cholesterol-containing membranes [9,21]. The expression of a non-haemolytic PLY might make serotype 1 bacteria less damaging and harmful for the host, and possibly helps to explain why serotype 1 diseases are associated with lower case fatality rates compared to other pneumococcal infections [5]. However, not all serotype 1 pneumococci carry the non-haemolytic PLY [21], and further studies are clearly required to prove or disprove this hypothesis.

A recent study compared isogenic mutants in the D39 background and showed that mutants expressing allele 5 PLY exhibited a medium-level virulence compared to bacteria expressing haemolytic PLY or lacking any toxin expression [48]. Taken this fact together with epidemiological data might indicate that cytolytic activity is not an essential characteristic of pneumococci for being virulent. Non-cytolytic properties of PLY are at least similarly important. Moreover, the different activities of PLY might be even less essential for secondary pneumococcal pneumonia following influenza virus infection [49].

We suggest that MLST306 serotype 1 might actually take advantage of expressing a non-haemolytic toxin variant. The expression of allele 5 PLY enables serotype 1 pneumococci to avoid the detection by inflammasomes and to minimize the production of key pro-inflammatory cytokines. We speculate that the evasion of inflammasome-dependent detection by serotype 1 contributes to its ability to invade sterile compartments. Of note, expression of haemolytic PLY has additionally been shown to be required for the detection of pneumococcal peptidoglycan by NOD2 and DNA by a STING dependent pathway, respectively [47,50–52]. Avoiding recognition by those pathways might thus also add to the relative particularity of serotype 1 pneumococci. Potentially in line with this assumption, serotype 1 pneumococci appear to be weak inducers of *Il1b* mRNA (Fig 1I). Moreover, it is likely that other PLY-independent characteristics of this strain such as e.g. capsule or cell wall composition influence the interaction with the host´s innate immune system. For example, we recently showed that serotype 1 bacteria differ to other serotypes in their binding to the C-type lectin receptor Mincle [53].

Taken together, we demonstrate that different pneumococcal serotypes differentially activate the NLRP3 inflammasome in human lung tissue, and that important serotype 1 and 8 clones evade recognition by this central innate immune sensing mechanism. It will be important to further dissect the contribution of this innate immune evasion to the pathogenic potential of these globally emerging bacterial strains.
